# Correspondence Between Cognitive and Audiological Evaluations Among the Elderly: A Preliminary Report of an Audiological Screening Model of Subjects at Risk of Cognitive Decline With Slight to Moderate Hearing Loss

**DOI:** 10.3389/fnins.2019.01279

**Published:** 2019-12-10

**Authors:** Alessandro Castiglione, Mariella Casa, Samanta Gallo, Flavia Sorrentino, Sonila Dhima, Dalila Cilia, Elisa Lovo, Marta Gambin, Maela Previato, Simone Colombo, Ezio Caserta, Flavia Gheller, Cristina Giacomelli, Silvia Montino, Federica Limongi, Davide Brotto, Carlo Gabelli, Patrizia Trevisi, Roberto Bovo, Alessandro Martini

**Affiliations:** ^1^Department of Neurosciences, University of Padua, Padua, Italy; ^2^Complex Operative Unit of Otolaryngology, Hospital of Padua, Padua, Italy; ^3^Regional Center for the Study and Treatment of the Aging Brain, Department of Internal Medicine, Padua, Italy; ^4^Institute of Neuroscience, National Research Council, Padua, Italy

**Keywords:** cognitive decline, hearing loss, Italian Matrix Sentence Test, logatomes, signal-to-noise ratio, slope, speech in noise, screening

## Abstract

Epidemiological studies show increasing prevalence rates of cognitive decline and hearing loss with age, particularly after the age of 65 years. These conditions are reported to be associated, although conclusive evidence of causality and implications is lacking. Nevertheless, audiological and cognitive assessment among elderly people is a key target for comprehensive and multidisciplinary evaluation of the subject’s frailty status. To evaluate the use of tools for identifying older adults at risk of hearing loss and cognitive decline and to compare skills and abilities in terms of hearing and cognitive performances between older adults and young subjects, we performed a prospective cross-sectional study using supraliminal auditory tests. The relationship between cognitive assessment results and audiometric results was investigated, and reference ranges for different ages or stages of disease were determined. Patients older than 65 years with different degrees of hearing function were enrolled. Each subject underwent an extensive audiological assessment, including tonal and speech audiometry, Italian Matrix Sentence Test, and speech audiometry with logatomes in quiet. Cognitive function was screened and then verified by experienced clinicians using the Montreal Cognitive Assessment Score, the Geriatric Depression Scale, and further investigations in some. One hundred twenty-three subjects were finally enrolled during 2016–2019: 103 were >65 years of age and 20 were younger participants (as controls). Cognitive functions showed a correlation with the audiological results in post-lingual hearing-impaired patients, in particular in those affected by slight to moderate hearing loss and aged more than 70 years. Audiological testing can thus be useful in clinical assessment and identification of patients at risk of cognitive impairment. The study was limited by its sample size (CI 95%; CL 10%), strict dependence on language, and hearing threshold. Further investigations should be conducted to confirm the reported results and to verify similar screening models.

## Introduction

Aging is epidemiologically associated with increasing prevalence rates of hearing loss and cognitive decline (Lin et al., 2011a, b; Peracino and Pecorelli, 2016). This association has been widely reported in the literature since a study by Herbst and Humphrey (1980). Even if the rational implications are debated in the literature and a causal relationship remains far from being proven, the audiological and cognitive assessment among elderly still remains a key component of a comprehensive and multidisciplinary approach to determining potential frailty (Panza et al., 2015).

Interest in this topic (Thomson et al., 2017) has grown in the last decade mainly due to demographic and sociocultural changes (Homans et al., 2017; Limongi et al., 2017: Livingston et al., 2017; Rooth, 2017); difficulties in treating neurodegenerative disorders, which has led to increased research into modifiable risk factors (Kostoff et al., 2017; Vos et al., 2017); the potential effects of peripheral hearing loss (Lin et al., 2011b; Lin et al., 2013; Wayne and Johnsrude, 2015; Deal et al., 2017); and rehabilitative as well as economical roles of digital devices (such as hearing aids, cochlear implants, over-the-counter amplification products) (Jorgensen and Messersmith, 2015; Cosetti et al., 2016; Nguyen et al., 2017). Additionally, the growing use of new tests and technologies has globally promoted reassessment of cognitive functions (Shen et al., 2016). The clinical distinction between central and peripheral hearing loss facilitates estimation of different contribution rates (Parham and Kost, 2017), even if, in practice, it is very difficult to quantify the relative portions, especially among older adults. In fact, there are different types of hearing impairment, as well as different types of cognitive decline, and these can be differently correlated or respond differently to treatments.

Defining the specific role of audiology in this context is important, and it is necessary to assess, design tests, and determine range limits in aging populations. To this end, the signal-to-noise ratio (SNR) can be used to define different auditory conditions and it is a good candidate for evaluating the contribution of auditory status to high cognitive functions (Panza et al., 2015; Costa et al., 2016; Livingston et al., 2017; Parham and Kost, 2017; Thomson et al., 2017; Jayakody et al., 2018). The Italian Matrix Sentence Test allows adaptive examination and results in noise (Puglisi et al., 2015). In addition, in selected cases, use of logatomes can elucidate auditory functioning in attention and working memory (Muhler et al., 2009; Moradi et al., 2014; Schubotz et al., 2016).

Thus, we performed a prospective study with cross-sectional measurements to evaluate the use of those tools for identifying older adults affected by hearing impairment and at risk of cognitive decline. In addition, the study aims to contribute in establishing age-appropriate reference intervals of specific tools in different hearing and cognitive impairments.

## Materials and Methods

One hundred sixty-six subjects were screened for inclusion criteria in this study between 2016 and 2019: adults older than 65 years and native Italian speakers with or without hearing loss or cognitive decline were enrolled as cases. The control group consisted of 20 young students (10 females) (median age 21, range 18–42 years) with normal hearing. Younger adults were also included to normalize reference ranges for different ages and to estimate the statistical power, also by comparing with literature on the same topic.

Exclusion criteria were lack of cooperation, life-threatening diseases, psychotropic therapies, history of disabling cardiovascular diseases, ictus or other potential life-threatening conditions, myocardial infarction, transient ischemic attacks or stroke, familiarity of neurodegenerative processes, or in advanced stages of disease. Patients with a history or family history of neurodegenerative diseases were excluded to avoid the contribution of early-onset diseases, which typically have a genetic etiology (Giau et al., 2019). Additionally, adults with hearing aids or cochlear implants were also excluded to avoid interference of digital devices with the acoustic properties of the auditory stimuli, which could significantly modify the SNR (Gallo and Castiglione, 2019). Patients with hearing impairment of genetic origin and those with severe to profound hearing losses were also excluded. These exclusion criteria were comparable to those of previous reports of investigations of mild cognitive impairment (MCI) among the Italian population to yield homogeneous and comparable data for cutoff settings (Bovo et al., 2007; Conti et al., 2015; Santangelo et al., 2015).

### Audiological Assessment

An audiological evaluation was carried out by clinicians and technicians with proven experience in the management of hearing loss. Testing included otoscopy, tonal and speech audiometry in quiet, using disyllabic words, the Italian Matrix Sentence Test (OLSA test), with an adaptive SNR (in dB) at which subjects can recognize 50% of the speech material in noise of 65 dB sound pressure level (SPL) in an open set with a frontal speaker (0 degrees) at 1 m distance and the slope in dB/% for speech discrimination in noise. The audiometric tests were randomly administered in a soundproof booth using the Noah and Otosuite software with standardized Italian language acoustic materials provided by Otometrics (a division of Natus in Taasrup, Denmark) and HörTech (Oldenburg, Germany).

The following data were obtained in a quiet environment: (1) The pure tone average (PTA) value at 0.5, 1, 2, and 4 kHz, in dB HL (PTA dB HL); (2) the signal/speech recognition threshold (SRT), in dB SPL, at which subjects could identify 50% of disyllabic words; (3) speech audiometry with logatomes. The latter were phonetic units without meaning, which can have the following consonant–vowel construction: CVC, VCVC, CVCV. In the Italian language, these structures can be assimilated to form pseudo-words or words non-words and, in general, presented in the following form: VCV or VCVV. Each list used in the present study consisted of 10 randomly selected logatomes. The choice to use this type of speech audiometry aims to minimize the mnemonic effort of the subjects: the speech intelligibility tends to differ between logatomes and words because they are not supported by semantic and long-term memory (Pisoni, 1996; Chan and Alain, 2018). The average of the intrasubject differences has been estimated between 5 and 20% (in favor of words) of recognized signals, even in the best hearing conditions. To identify different groups and carry out statistical analysis, the difference (hereinafter Log. Diff.) between the maximum intelligibility score, in%, for familiar words and the maximum intelligibility score, in%, for logatomes (i.d. maximum speech score with words – maximum speech score with logatomes) was calculated, and the cutoff was arbitrarily set to 10%, as suggested by a previous report (Moradi et al., 2014) to identify subjects potentially out of reference ranges. This allowed correlation among the Log. Diff. and attention or working memory, particularly if this was difficult among older adults (Santangelo et al., 2015). As mentioned before, the difference in percentage of identification of logatomes and words can be set within 10%, at the maximum comfortable level of acoustic signal in dB SPL for normal-hearing adults without cognitive decline. Thus, the maximum speech discrimination score for disyllabic words in a quiet environment is very close to that for logatomes in quiet, independent of differences in intensity levels. Therefore, the difference in a quiet environment should be less than 10%, even at different dB levels (Moradi et al., 2014).

The OLSA test (HörTech) is a versatile examination that is essentially structured into 20 randomized lists of five-word sentences (Houben et al., 2014), semantically unpredictable and administered after a training session to minimize the learning curve. The test can be useful in evaluating a wide range of conditions and treatments: congenital hearing loss, presbycusis, neuropathies, and auditory rehabilitation with hearing aids or cochlear implants. The test yields three main measurements: (1) the SNR, in dB, at which the subject recognizes 50% of the presented words, even if in different sentences (SNR-SRT); (2) the slope of the discrimination function at SRT (Slope) in percentage (%/dB); and (3) the intelligibility percentage score, in terms of estimated accuracy in understanding whole sentences. The global test in itself is an automated version of the synthetic sentence test, termed the synthetic sentences identification test (SSI) (King-Smith and Rose, 1997; Kaernbach, 2001; Buss et al., 2009; MacPherson and Akeroyd, 2014; Kollmeier, 2015). The novelty and the strength of the test are the speech noise material, with features inspired by ICRA noise, and its simplicity in performing adaptive exams (Dreschler et al., 2001; Wagener et al., 2006; Meister et al., 2013; Akeroyd et al., 2015). Reviewing reference ranges and standard deviations for the Italian language of the OLSA test, published by Puglisi et al. (2015), allowed determination of different levels to identify at-risk patients. The cutoff of the SNR dB (SRT) among the elderly was set to – 0.4 dB based on a reference mean level of −6.7 plus 9 standard deviations (3 SDs include 100% of samples divided by age). Thus, older adults are 6–12 SDs away from normal hearing and hearing in a younger population; the slope cutoff was set to 9.4% (reference level of 13.3% minus 3 SDs) (Puglisi et al., 2015). Audiological tests were conducted blinded to the cognitive status.

### Cognitive Assessment

Subjects participated in a screening phase. Cognitive function was screened using the Montreal Cognitive Assessment (MoCA, adjusted for education) score: the presence of mild cognitive dysfunction was suspected when the final score was less than 26. Further clinical investigations were carried out by specialists in the management of dementia and cognitive decline (neuropsychologists and geriatricians). In addition, previous clinical data, including neuroimaging, and history available on digital archives were reviewed by medical doctors for definitive assessment. The evaluation was extended in selected cases to confirm screening results, and it included the forward and backward digit span test, drawing tests (clock, cube, house, pentagons), the trail-making test, the digit symbol substitution test, and the Direct Assessment of Functional Status (DAFS) for measuring Alzheimer’s disease severity (Zanetti et al., 1998). The DAFS also helped in defining preservations of daily activities. The Geriatric Depression Scale (GDS) (30 items, long form) was used to screen depressive symptoms.

Mild cognitive decline was confirmed when daily activities were preserved, and cognitive decline was defined when these were compromised based on the diagnostic criteria reported in the literature and the DSM-V. Nevertheless, prior to further investigations and final diagnosis, patients were considered as only being at risk of, or likely to have MCI, because the screening model should be not considered diagnostic.

Participants with good cognitive performance and some degree of hearing loss (if any) in few frequencies, but without disabling hearing impairment, were defined healthy: this group was named the “healthy aging.”

The cutoff MoCA score for risk of cognitive impairment has been varied (26, 24, 22) to verify differences in specificity and sensitivity (Davis et al., 2015). In addition, for further analysis, the scores for attention and working memory were set to 4.8 (<5) in the digit span test and MoCA (Moradi et al., 2014; Santangelo et al., 2015), whereas the long-term memory score was set to 2.80 (<3). These scores are the approximate normal/average reference values, reduced by 2 SDs (Moradi et al., 2014; Conti et al., 2015; Davis et al., 2015; Santangelo et al., 2015; Castiglione et al., 2016; Kujawski et al., 2018; Siciliano et al., 2019). Subjects with clinical indications were selected for hearing aid prescription, cochlear implantation surgery, or further cognitive investigations.

### Statistical Analysis

Statistical analysis was performed through Microsoft Office Excel 2016 (Redmond, WA, United States) with data analysis plug-ins and MedCalc (Ostend, Belgium). The following tests were used: Student’s *t*-test, Mann–Whitney test for independent samples (unpaired), Fisher’s exact test and relative risk in 2 × 2 tables, analysis of variance (ANOVA), coefficient of Pearson correlation, and multiple regression analysis. Results were considered significant when *p* < 0.01.

### Ethical Issues

This study was carried out in accordance with the recommendations of national and international guidelines. All subjects gave written informed consent in accordance with the Declaration of Helsinki. The protocol was approved by the local ethical committee of the University Hospital of Padua. The research was funded by the University of Padua, the Azienda Ospedaliera di Padova, Cochlear Italia Srl, and Amplifon SpA, as part of the main project PRIHTA-IDECO 2013, approved by the local ethics committee in 2016 (n° AO0379-0059267-CE3361/AO/14).

## Results

One hundred sixty-six subjects were included in this study between 2016 and 2019, at the clinic of Otorhinolaryngology of the University Hospital of Padua, as part of the PRIHTA-IDECO 2013 project. Forty-three subjects were excluded because of the degree of hearing loss: severe to profound hearing-impaired patients had a speech recognition score below 50%. The other 123 subjects were then divided into two groups: the cases included 103 subjects (51 females) older than 65 years (median age 71, range 65–93 years). The control group consisted of 20 young students with normal hearing (10 females, median age 21, range 18–42 years). Hearing loss among the cases was classified according to the criteria of the World Health Organization. Among the cases were identified 17 (16.50%) subjects with normal hearing, 21 (20.40%) with slight loss of hearing, and 65 (63.10%) with mild to moderate hearing loss, of which four were with characteristics very close to severe losses. There is also a correlation between hearing loss and age and cognitive decline, so that subjects with better hearing and better cognitive performance are grouped in the age group between 65 and 70 years.

A summary of the characteristics of the cases and controls is given in Table 1. Distributions by PTA dB HL, age, and MoCA scores are reported in Table 2. No differences were found between the left and the right ear, even if the right ear showed slight advantages in discrimination probably due to interhemispheric dominance (*p* > 0.05). The control group allowed normal range adjustment for testing among the elderly and confirmed the feasibility of the test in different audiological and cognitive conditions.

**TABLE 1:** Major clinical findings in the control group (young adults with normal hearing) and older adults (>65 years, with normal to moderate hearing impairment).

			**Controls: 20 (10 F)**			***P value***	**Cases: 103 (51 F)**		
			**Mean**	**Median**	***SD***	***t*-test**	**Mean**	**Median**	***SD***
		Age (years)	23.20	21	6.22	<*0.001*	72.62	71	7.03
Hearing		PTA dB HL	8.87	10	4.33	**<*0.001***	32.77	33.75	15.26
		SRT	13.50	15	5.20	**<*0.001***	36.41	35	15.49
		SNR	–6.88	-6.75	0.96	**<*0.001***	1.42	−0.3	6.71
		Slope in Noise	15.03	16	4.53	**<*0.001***	10.76	10	4.18
		Intelligibility in noise (% of S.I.)	55.48	56	2.9	**<*0.001***	50.13	52	7.73
		Logatomes (difference in%)	3.00	2.5	3.35	**<*0.001***	11.31	10	12.43
Cognition	MoCA	Tot. MoCA (score)	29.70	30	0.66	**<*0.001***	25.15	26	3.89
		Attention and Working Memory	5.80	6	0.41	**<*0.001***	4.83	5.00	1.56
		Long-term memory	4.35	4.00	0.67	**<*0.001***	2.61	3.00	1.80

*F, females; MoCA, Montreal Cognitive Assessment; S.I., Intelligibility (%) of sentences and words in noise around the SNR value; SNR, signal-to-noise ratio; SRT, speech/signal recognition threshold; SD, standard deviation. Bolded values indicate significant results.*

**TABLE 2:** Age distribution of hearing and cognitive results.

***Age (years)***	***18–45 (age in years)***	***65–69 (age in years)***	***70–79 (age in years)***	**>*79 (age in years)***
n°(of cases)	**20**	**39**	**44**	**20**
*MoCA (score)*	29.7	27.41	25.15	20.75
*Slope (%)*	17.3	12.8	10.65	6.95
*SNR (dB)*	−7	−1.5	1.75	7.89
*Intelligibility in Noise (%)*	57.9	53	51.25	41.65
PTA (0.5-1-2-4-kHz)	8.62	26.82	33.15	43.5
*SRT (%)*	9	30.9	36.02	48
*Log. Diff. (%)*	3	5.5	11.53	22.12
*GDS (score)*	n.a.	5.48	5.09	5.05
*Long Term Memory (score)*	4.5	3.8	2.6	2
*Attention (score)*	5.8	5.8	5.2	3.5
*Log Intelligibility (%)*	97	77.14	80.6	57.5
*MCI (n*° *of Cases)*	0	6	21	17

*MoCA, Montreal Cognitive Assessment score (mean); Slope, percentage (mean); SNR, signal-to-noise ratio in dB (mean); S.I., sentence intelligibility in noise, percentage of correct sentences and words (mean); PTA, pure tone average at 500, 1,000, 2,000, and 4,000 Hz (mean in dB HL); SRT, speech/signal recognition threshold, mean in dB SPL of thresholds required to recognize 50% of words in quiet; Log. Diff., difference in% between words intelligibility and logatomes intelligibility (mean); GDS, Geriatric Depression Scale (long form); Memory (long-term), mean of cognitive tests; attention, mean of cognitive tests; MCI, mild cognitive impairment (number of diagnosis for each group); n.a., not applicable. Bolded values indicate significant results.*

As expected, significant differences were found between cases and controls in all variables, except for sex. Significant differences were also shown between patients with signs of MCI and those with normal cognitive function in terms of PTA, speech in noise, and the slope of psychometric functions. However, in the present study, GDS results are not significant. Among cases, two subgroups were defined: the healthy aging subgroup with a MoCA score > 26, adjusted for education, without signs of decline in cognitive tests or neuroradiological findings, and the MCI subgroup, identified as individuals at risk of MCI through the MoCA test (Table 3), and defined by cognitive scores < 26 adjusted for education (Conti et al., 2015). To give comparable results, *t*-tests and Mann–Whitney tests are reported, as many variables can be considered non-parametric, even when values appear parametric; nevertheless, there were no differences between the groups for either test at α = 0.01 (Table 3).

**TABLE 3:** Comparisons between the healthy aging group and subjects with or at risk of mild cognitive impairment (MCI).

			**103 Cases (51 F)**						
			**At risk of MCI**						
			**59 Cases (37 F)**				**44 Cases (14 F)**		
			**Healthy aging**				**MCI^∗^ (screened by MoCA)**		
			***Mean***	***Median***	***SD***	***p-value***	***Mean***	***Median***	***SD***
		Age	69.34	68	5.61	**<*0.001***	76.95	78	6.44
Hearing		PTA dB HL	27.53	23.75	18.41	**<*0.001***	49.52	41.25	19.61
		SRT	25.23	25	10.89	**<*0.001***	47.30	45	17.75
		SNR dB (SRT)	–0.83	−3.0	6.07	**<*0.001***	4.56	2.9	6.49
		Slope in Noise	12.43	12	3.84	**<*0.001***	8.67	8	3.98
		Intelligibility in noise	52.65	54	6.26	**<*0.001***	46.51	48.5	8.81
		Logatomes (difference in%)	6.16	0	9.97	**<*0.001***	18.78	15	13.23
Cognition	MoCA	Tot. MoCA (score)	27.74	28	1.29	**<*0.001***	21.67	22	3.54
		Attention and Working Memory	5.70	6	0.57	**<*0.001***	3.44	4	1.59
		Long-term memory	3.3	4	1.48	**=*0.001***	1.47	1	1.68
	GDS		5.22	4	3.39	***N.S. (p* = *0.8)***	5.28	4	4.34

*^∗^F, females; MCI, mild cognitive impairment as determined by Montreal Cognitive Assessment (MoCA) scores and confirmed with subsequent investigations by specialists; PTA dB HL, The average of pure tone audiometry around the most important frequencies for speech understanding, that is, 500, 1,000, 2,000, and 4,000 Hz. The *p*-values are obtained by Student’s *t*-test and the Mann–Whitney test in case of non-parametric variables; GDS, 30-item geriatric depression scale; N.S., not significant; SD, standard deviation. Bolded values indicate significant results.*

**FIGURE 1:**
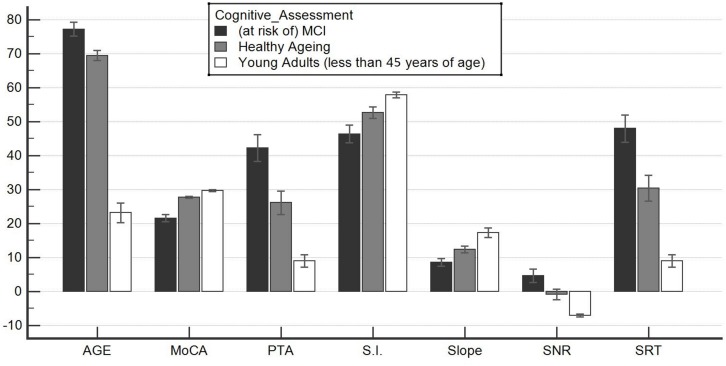
Significant differences among the healthy aging group and the at-risk mild cognitive impairment (MCI) group. This graph shows an analysis of variance for parametric and non-parametric variables. All comparisons were significant; thus, the groups identified through the Montreal Cognitive Assessment (MoCA) test (healthy aging and at risk of MCI) were in fact distinct. They also differed significantly in terms of their audiological profile, although they were not differentiated on the basis of their hearing ability. Healthy aging individuals were also statistically distinct from younger subjects. This suggests cognitive and auditory difficulty/fatigability that physiologically accompanies advancing years. These data, already reported in the literature, were not documented by audiometric comparisons that allow determination of whether loss is paraphysiological. In this way, a healthy aging subject is clearly defined with certain audiometric characteristics: (1) the ability to recognize a signal with a signal-to-noise ratio (SNR) < 0; (2) the pure tone audiometry of less than 30 dB HL; (3) an SRT at rest of less than or equal to 30 dB SPL; (4) a difference between max% words in quiet and logatomes of less than 10; and (5) a slope of psychometric function of greater than 10%. S.I., speech intelligibility in noise. See also Tables 1–4.

Comparison of the three groups revealed significant differences, with increasing *p*-value from CONTROLS to MCI patients. ANOVA revealed significant differences among these two groups (*p* < 0.001), and the results are reported in Figure 1.

The study of correlation revealed a moderate to strong coefficient between SNR and MoCA scores. Other correlations and multiple regression analysis are shown in Table 4. Age showed the highest negative correlation with cognitive results, but it should be noticed that groups were arbitrarily divided by age and that the MoCA scores were adjusted for years of education. Thus, in higher age groups, the MoCA score could be adjusted for age because after 78 years, participants were unlikely to have been attending educational and recreative programs during childhood, as compared to the new 65-year-old subjects of coming decades (Kujawski et al., 2018). Nevertheless, it should be noticed that the SNR showed the highest negative correlation among the audiological testing results and that the multiple regression analysis reached an *R*^2^ of 0.477. These results suggested a strong correlation with cognitive impairment particularly when the variables were considered together. It could be hypothesized that the cognitive results and the audiological tests, when considered together, can explain approximately 50% of the final score (and its variance) obtained for each subject.

**TABLE 4:** Regression analysis and coefficient of correlation of the Montreal Cognitive Assessment (MoCA) score with audiological results.

	***Age***	***Slope***	***SNR***	***S.I.***	***PTA***	***SRT***	***Log. Diff.***
Coeff. Corr.	−0.670	0.521	−0.544	0.563	−0.469	−0.472	−0.442
*R*^2^	0.449	0.272	0.296	0.317	0.220	0.223	0.195
*p*-_*value*_	<0.001	<0.001	<0.001	<0.001	<0.001	<0.001	<0.001

**Multiple Regression (Dependent Y MoCA)**							
***R*^2^ adjusted**	**Multiple corr. coeff.**	**F-ratio**			***P*-value**		
**0.477**	0.716	14.287			**<0.0001**		

*PTA, pure tone average; S.I., sentence intelligibility in noise; SNR, signal-to-noise ratio; SRT, speech/signal recognition threshold; Log. Diff., different discrimination percentages between logatomes and words. Bolded values indicate significant results.*

In Tables 5, 6, we show the different sensitivity and specificity values for identifying different levels of cognitive impairment. The associated receiver operating characteristic (ROC) curves are illustrated in Supplementary Figure S1. The ROC curves show the predictive results of the screening model at various cutoff values. Different ROC curves are shown to compare sensitivity and specificity of different variables for identifying patients with cognitive decline, as represented by a MoCA score < 26 and subsequent confirmed diagnosis of MCI. The SNR reaches highest rates of sensitivity and specificity (Supplementary Figure S1). This curve allows intuitive prediction of the sensitivity and specificity of a test for any value obtainable from the test; various tests are also compared to assess which one has the best chance of identifying true positives and true negatives. The audiological evaluation is comparable to those attributed to the MoCA test. Age had suitable characteristics but a lower specificity. These data can only be considered preliminary as a possible screening model that can promote a multidisciplinary approach because the patient often does not know the origin of his own difficulty and may therefore seek help from various specialists. Given that this is proposed as a screening model, high sensitivity is crucial, but subsequent diagnostic confirmation is required.

**TABLE 5:** Sensitivity and specificity of different audiological results for identifying cognitive impairment.

**MCI**					
	**TP**	**FP**	**TN**	**FN**	**tot**
SNR ≥ -0.4 dB	36	18	41	8	103
Slope ≤ 9.4%	26	9	50	18	103
Logatomes ≥ 10%	38	20	39	6	103

	**SNR ≥ -0.4 dB**	**≤ 9.4%**	**Log. Diff. ≥ 10%**		

Sens.	0.818	0.743	0.864		
Spec.	0.695	0.735	0.661		

*MCI, mild cognitive impairment; Spec, specificity; Sens, sensitivity; SNR, signal-to-noise ratio; tot, total; TP, true positive; FP, false positive; TN, true negative; FN, false negative.*

**TABLE 6:** Combining Log.Diff.-Slope-SNR (passing two or three criteria for the screening model) increases sensitivity and specificity of audiological screening.

**MCI < 26**					
	**TP**	**FP**	**TN**	**FN**	**tot**
	35	15	44	9	103

**SNR ≥ -0,4 dB AND/OR**				***CI 95%***	
**Slope ≤ 9.4% AND/OR**					
**Logatomes Diff. ≥ 10%**					

Sens.	0.795			0.647–0.902	
Spec.	0.746			0.616–0.850	

*Logatomes Diff., the difference in terms of percentage of recognized words/logatomes among the maximum achievable scores in speech audiometry in quiet and the maximum achievable score in logatome audiometry in a quiet environment; MCI, mild cognitive impairment; SNR, signal-to-noise ratio; Sens, sensitivity; Spec, specificity, tot, total; TP, true positive; FP, false positive; TN, true negative; FN, false negative.*

As mentioned before, the cases were subsequently divided into two subgroups to compare auditory and cognitive functions. In 59 cases, participants were healthy, with good cognitive performance and some degree of hearing loss (if any), but without disabling hearing impairment; this group was named the healthy aging. In contrast, 44 participants were classified as at risk of MCI, showing signs of cognitive impairment. Further cognitive test results and neuroimaging (when possible or indicated) were used to confirm the MoCA results indicative of MCI. Eight of these 44 patients (18.18%) were affected by early-stage vascular cognitive impairment with suggestive neuroradiological findings, 22 (38%) showed memory impairment in multiple domains on cognitive tests, and 14 (38%) were initially classified as having an uncertain or borderline diagnosis (Table 3) because of inconclusive diagnostic results or incongruent findings. Eight of the 44 patients (18.18%) were subsequently correctly reevaluated, and MCI in multiple non-amnestic domains was confirmed (Supplementary Table S1). The remaining four patients were designated as patients with MCI affecting multiple amnestic domains. Thus, 63.64% (28 of 44) of the screened populations were defined as having amnestic multiple domain MCI, agreeing with reports in the literature. Among the healthy aging group, 9 (of 59) subjects were reevaluated because of inconclusive results probably related to transient anxiety or low attention in performing the test or because they were too fatigued to complete the investigations. Therefore, finally, 23 (of 103) patients required reinterpretation and reanalysis of results.

The relative risk and two-tailed Fisher’s exact test for different clinical settings and cutoff values are presented in 2 × 2 tables in Supplementary Material (Supplementary Table S1).

## Discussion

**FIGURE 2:**
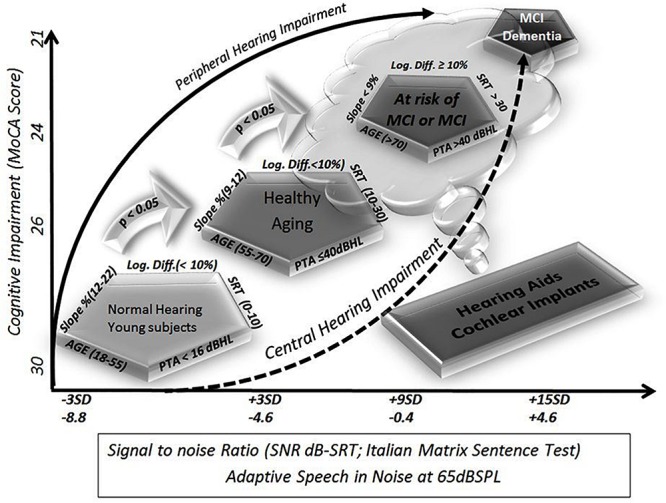
Schematic representation of cognitive decline as defined by different audiological tests. The diagrams summarize the present study. On the *y*-axis, the results of the Montreal Cognitive Assessment (MoCA) score are shown as the average result of the groups shown in the figure as pentagons (ideally defined by five variables, four audiological parameters plus age). On the *x*-axis, there are reference levels for the signal-to-noise ratio (SNR) available in the literature, such as a test for matrices with speech in noise sentences. The test was recently chosen for diffusion in clinical practice in Europe for its easy automatic execution and because it yields easily comparable values regardless of age and language. Starting from the available and official reference levels, and moving in steps of 3 standard deviations, so as to include 100% of the study population, it was possible to identify four different populations, which will by definition be statistically significantly different. The groups used in this study fall exactly within those theoretical values: young subjects, elderly but healthy subjects, and elderly subjects suffering from mild cognitive decline either because they were diagnosed or because they are rated using cognitive tests, and finally, at the opposite extreme, patients suffering from dementia who were so severely affected that they were not able to complete the tests.

In this preliminary study, we found a significant correlation between cognitive scores and SNR, slope, and logatome intelligibility. Even if the MoCA tests should be considered purely indicative and not diagnostic, the results were verified by clinicians experienced in the management of neurodegenerative processes affecting high cognitive functions. To verify the feasibility of a screening model involving audiological tests, the MoCA score was chosen as representative of real-life cognition. Audiological tests were conducted blinded to the cognitive status, and the results were found to correlate statistically significantly. Using these quantitative and semiquantitative parameters, auditory functioning could be assessed, and the risk for cognitive decline can be suspected for further investigations in selected patients. In addition, it is possible to define a reference area or zone, delineated by all of these measures, including individuals with similar characteristics (Figure 2). In other words, patients at risk of developing cognitive decline can suffer from a reduced ability to cope with living in a noisy world (auditory frailty). This disadvantage points to the type of rehabilitative action that might reduce the risk of cognitive decline or frailty. The tests might define different contributions to cognitive fatigue by peripheral and central hearing losses, at different ages, thus reflecting a more realistic pattern for longitudinal studies. Even if all tests require cortical efficient functioning, it can be hypothesized that tonal audiometry is one of the simplest tests that a patient can actively perform, and, consequently, it entails poor perceptual involvement of the central neuronal stations. Conversely, progressive involvement of cortical structures is more evident in speech discrimination and in sentence discrimination in noise.

Due to the correlation between hearing loss, aging, and cognitive decline, the results also reflect a pattern of involution, thus the screening model is more congruent for patients with slight to moderate hearing loss among 65–75 years of age (Table 1). These results could suggest different contributions of peripheral and central hearing loss to global cognitive functions during aging, and it should suggest range limits for treatments to restore hearing function with hearing aids or cochlear implants.

In the last decade, there has been a great deal of interest in and discussion about the relationship between cognitive decline and hearing loss (Lin et al., 2013; Bernabei et al., 2014; Wayne and Johnsrude, 2015; Hewitt, 2017; Rutherford et al., 2018; Stern and Hilly, 2018; Uchida et al., 2018). Studies on the aging brain have shown that cognitive functions tend to (para-)physiologically decline with advancing years (Ciorba et al., 2011; Ding et al., 2016). In addition, 14.3% of the population over 65 years of age is affected by central auditory processing disorders (CAPD). Older individuals with CAPD seem to be more likely to suffer from dementia than those who are not affected (Martini et al., 2014; Fortunato et al., 2016). The decline can be highlighted in circumstances requiring greater cognitive effort and stressful conditions, such as speech discrimination of words or logatomes in noise or in quiet (Martini et al., 1988; Strauss et al., 2015; Gobara et al., 2016; Taitelbaum-Swead and Fostick, 2016; Bae et al., 2018). Competitive stimuli should be the most sensitive for detecting cognitive efforts or difficulties among elderly people (Pronk et al., 2013). The SNR is a well-known semiquantitative, a parameter, which is useful in the audiological assessment of various conditions of clinical interest, although its role in evaluating cognitive functions is less clear (Pronk et al., 2013; Helfer, 2015; Meister, 2017).

Young subjects have better discrimination than individuals older than 65 years. Furthermore, the use of logatomes can help identify patients with preserved long-term and semantic memory, even though they are at risk of cognitive decline because of impairments in attention and working memory. When semantic content is lacking, speech discrimination requires additional cognitive effort, and subjects with better working memory capacity demonstrate superior performance in the identification of the speech signals (Kim and Oh, 2013). This has been confirmed by neuroimaging studies that show the involvement of both the prefrontal and auditory cortex for the correct identification of ambiguous phonemes (Moradi et al., 2014). In addition, confusion matrices among logatomes (AFA/ATA/ASA, AGIA) can detect some types of cognitive disorders among children (Sundstrom et al., 2018), although their role in the audiological and cognitive assessment among older adults is not as clear (Chen and Cowan, 2009). Even if hearing-impaired patients show some disadvantages in speech recognition, the intrasubject differences in identifying words and pseudo-words should be in the 5–20% range (10% ± 2 standard deviation) at the maximum comfortable level of stimulation in dB SPL (Carrat and Carrat, 1992; Apoux et al., 2001; Moradi et al., 2014; Schubotz et al., 2016). Speech audiometry with logatomes might allow identification of those patients who fall outside of this range (Tables 5, 6).

Even if it is not routinely performed, such extensive audiological assessment, including SNR, slope of functions, and logatomes, after 55 years of age might help in identifying patients at risk of cognitive decline prior to 65 years of age in the general population.

Nonetheless, due to the limitations of the present study, this report should be considered a tentative preliminary model of auditory screening in the elderly, which must be confirmed in further studies. To the best of our knowledge, reported results and cutoffs should be considered only among older adults with post-lingual mild to moderate hearing loss, and results are more consistent for people between 65 and 75 years of age. These subjects could take advantages from auditory rehabilitation through hearing aids for moderate hearing losses and cochlear implant for severe to profound hearing losses. Unfortunately, patients with severe to profound hearing loss presented limitations in performing all tests, therefore they required specific settings for subsequent investigations, and they were not comparable to elderly patients with better conditions. Consequently, specific populations, such as severe to profound hearing-impaired patients or subjects with cochlear implants and/or hearing aids, require different range references. Furthermore, protocol testing could be time-consuming especially in untrained centers, or among impaired elderly, thus the screening model might be unsustainable in routine or clinical practice. In addition, the fatigue of older adults might be a bias in the final assessment.

## Conclusion

In conclusion, our study highlights that, in the elderly, audiological assessment by means of SNR, slope of functions, and logatomes might help in further characterization of selected patients because of their correlation with age, high cognitive functions, and hearing loss. These tests might be helpful not only in the detection of hearing loss but also in early identification of the impairment of high cognitive functions. We also suggest that a stricter relation between audiological and neuropsychological assessment of selected patients should be established, and rehabilitation might benefit from such a comprehensive multidisciplinary approach.

Furthermore, our findings indicate that specific audiological results are representative of cognitive functions during aging, following an irregular progression, in part because they can be assimilated into cognitive tests and involve instructions, training, attention, time, accuracy, and cooperation. A mixed contribution of peripheral and central hearing impairment is involved in real life; nevertheless, these impairments contribute differentially during different stages of life. Irrespective of the causative explanation, hearing impairment presents disadvantages at any stage of life and can affect cognition as well as quality of life, increasing the frailty of subjects. Cognitive assessments should be accompanied by auditory testing.

It is important to remark limitations of the present research, thus there are no conclusive evidences on this topic and further investigations are required.

## Ethics Statement

This study was carried out in accordance with the recommendations of national and international guidelines, and ethical committee with written informed consent from all subjects. All subjects gave written informed consent in accordance with the Declaration of Helsinki. The protocol was approved by the Ethical Committee of the University Hospital of Padua.

## Author Contributions

AC supervised the all tests, collected the data, performed the statistical analysis, wrote the main part of the manuscript, and designed the figures and tables. MC helped in the audiological and cognitive assessment, collected the clinical data, and conducted the cognitive tests. SG and MG conducted the literature review, planned part of the work, and wrote part of the manuscript. EL, DC, FS, FG, EC, SG, SC, MP, and SD performed the audiological tests and collected the data. RB, PT, DB, FL, and CGa provided supervision and consultancy on clinical, audiological, and neuroimaging findings. SM and CGi assessed cognitive-communication deficits. AM is the principal investigator of the PRIHTA-IDECO 2013 project and supervised all of the steps of the research.

## Conflict of Interest

The authors declare that the research was conducted in the absence of any commercial or financial relationships that could be construed as a potential conflict of interest.

## Supplementary Material

FIGURE S1 Different receiver operating characteristic curves are shown, to compare sensitivity and specificity of different variables for identifying patients with cognitive decline, as represented by a Montreal Cognitive Assessment (MoCA) score < 26.

TABLE S1 Twenty-three patients with uncertain results were analyzed through the Fisher Exact Test to estimate the identification rate and relative risk of impairment in attention and working memory using logatomes.
