# Supplementary figures and images for: Correspondence Between Cognitive and Audiological Evaluations Among the Elderly: A Preliminary Report of an Audiological Screening Model of Subjects at Risk of Cognitive Decline With Slight to Moderate Hearing Loss
Source: Front Neurosci. 2019 Dec 10;13:1279. doi: 10.3389/fnins.2019.01279 (PMC6915032; doi:10.3389/fnins.2019.01279)

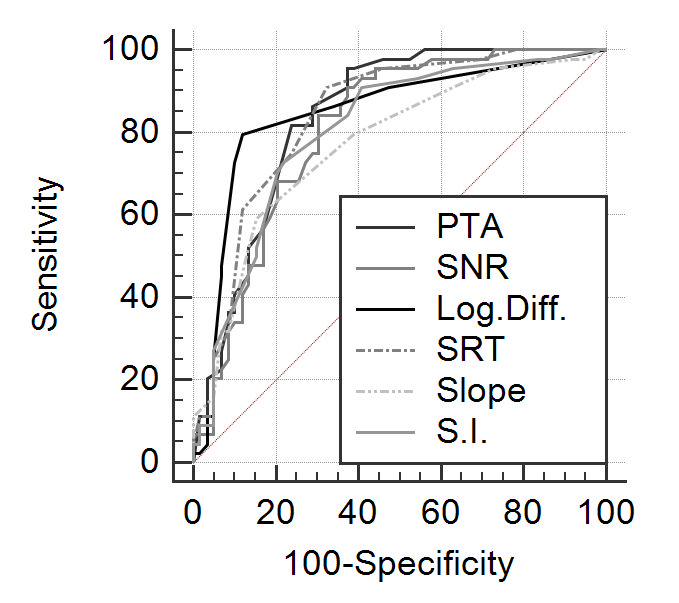

Supplement: FIGURE S1 — Different receiver operating characteristic curves are shown, to compare sensitivity and specificity of different variables for identifying patients with cognitive decline, as represented by a Montreal Cognitive Assessment (MoCA) score < 26. [file Image_1.TIF]
