# Supplementary material for: Correspondence Between Cognitive and Audiological Evaluations Among the Elderly: A Preliminary Report of an Audiological Screening Model of Subjects at Risk of Cognitive Decline With Slight to Moderate Hearing Loss
Source: Front Neurosci. 2019 Dec 10;13:1279. doi: 10.3389/fnins.2019.01279 (PMC6915032; doi:10.3389/fnins.2019.01279)
Supplement: TABLE S1 — Twenty-three patients with uncertain results were analyzed through the Fisher Exact Test to estimate the identification rate and relative risk of impairment in attention and working memory using logatomes. [file Table_1.DOCX]

**Supplementary material**

**TABLE S1** – Twenty-three patients with uncertain results were analyzed through the Fisher Exact Test to estimate the identification rate and relative risk of impairment in attention and working memory using logatomes.

| Events | Log. Diff. ≥ 10% | Log. Diff. < 10% |
| --- | --- | --- |
| Cognitive score in attention < 5 | 8 | 1 |
| Cognitive score in attention ≥ 5 | 5 | 9 |
| Fisher's exact test | p-value = 0.029 (2-tailed) |  |
| Relative Risk (95% CI) | 2.49 | 1.19 – 5.21 |
